# The Role of the Organization Structure in the Diffusion of Innovations

**DOI:** 10.1371/journal.pone.0126076

**Published:** 2015-05-15

**Authors:** Carlos Sáenz-Royo, Carlos Gracia-Lázaro, Yamir Moreno

**Affiliations:** 1 Centro Universitario de la Defensa. 50090 Zaragoza, Spain; 2 Institute for Biocomputation and Physics of Complex Systems. University of Zaragoza, 50009 Zaragoza, Spain; 3 Departamento de Física Teórica. University of Zaragoza, 50009 Zaragoza, Spain; 4 Complex Networks and Systems Lagrange Lab, Institute for Scientific Interchange, Turin 10126, Italy; Hong Kong Baptist University, CHINA

## Abstract

Diffusion and adoption of innovations is a topic of increasing interest in economics, market research, and sociology. In this paper we investigate, through an agent based model, the dynamics of adoption of innovative proposals in different kinds of structures. We show that community structure plays an important role on the innovation diffusion, so that proposals are more likely to be accepted in homogeneous organizations. In addition, we show that the learning process of innovative technologies enhances their diffusion, thus resulting in an important ingredient when heterogeneous networks are considered. We also show that social pressure blocks the adoption process whatever the structure of the organization. These results may help to understand how different factors influence the diffusion and acceptance of innovative proposals in different communities and organizations.

## Introduction

Innovation feeds social and economic progress and drives progress through enhanced efficiency [1–3], costs [4–6], and the quality of the products [7]. Innovation includes ideas, values, goods, services, technologies, processes, etc. that emerge and are disseminated both in markets, organizations and within social systems in general. Whether an innovation is effective or not depends on whether it can be assimilated by the socio-economic system it is designed for. This will be more or less complicated, depending on the setting in which it is disseminated. In markets, decisions to adopt the innovation and to commit the resources required to gain access to the product and use it for its intended purpose are normally taken by one and the same person. In organizations, on the other hand, the specialization of duties and distribution of authority and decision-making among various jobs mean that each stage in the innovation assimilation process may be carried out by different people, and this demands a greater level of coordination. The dissemination of innovation in social complex systems can therefore be described as a process of a succession of decisions regarding adoption forming groups of people, or markets, else it can be described as a process of internal diffusion throughout the implementation stage in organisations [8].

This paper examines the assimilation of innovation in social systems as a decentralized process. In such processes, early stage is key to the assimilation of innovation. At this early stage, either members unanimously accept the innovation or they continue with the status quo. When adoption and implementation are produced in separate stages a previous consensus in favour of innovation will simplify the subsequent implementation stage since the people who will need to be involved at a later date are the same ones that previously came out in favour of adopting it. The use of Agent Based Models (ABM) as a methodological tool for the study of social phenomena provides useful insights about the fundamental causal mechanisms at work in social systems [9–11]. The large-scale macroscopic effects of simple forms of microscopic social interaction are very often surprising and generally hard to anticipate, such as the diffusion of information, emergence of norms, coordination of conventions, or participation in collective action.

The traditional literature on the adoption and dissemination of innovation has been heavily influenced by the epidemiological models for the contagion of diseases and pathologies in human groups, adapted to the social environment (see, *e.g.*, [12], extensions like [13] and the subsequent introduction of the model in complex Networks [14–18]). Our work differs from the models based on epidemiological studies in several respects. First of all, people in the market o in the organization take binary decisions, positioning themselves either for or against an innovation, with relevant information reaching them from within their group of influence. Their stance is not firm until a favorable consensus is obtained. Meanwhile, the same person can change their position once or various times, *i.e.*, go from being in favor of the innovation to being against it and vice versa while there is someone in their environment that disagrees. In the likelihood of opting for one alternative or another, on the one hand we consider the positions in favor or against the innovation expressed by other people within the radius of influence of the person concerned (the influence is weighted according to the *social pressure* exercised by the group over the individual according to the culture that is accepted by the members); and on the other, individual value attributed to such innovation. The model presented here allows for interaction between individuals connected to learn at a rate *m*, which modifies the performance attributed to the status quo and innovation. This single interaction take place if the difference between partners’ performances is less than a threshold *ϵ*. The individual character of the performance recognizes the existence of diversity in skills and knowledge of the different people in the group. The skill and knowledge determine the performance of each individual with respect to the two alternatives, influencing its decision. In the more conventional models for disseminating innovation, however, adoption boils down to making decisions concerning use or purchase: those who adopt an innovation by, for instance, purchasing a new product for the first time, are then classified as adopters forevermore and only admit peer pressure conducive to the adoption and never in favor of the status quo.

Secondly, in this paper the structure of the network articulating the internal workings of the members of the organization or the market has a very important role. The network structure implies that the likelihood of a person being in favor of an innovation will vary depending on their position in the network. Furthermore, the network structure chosen will determine the final outcome of the initiation stage measured by the greater or lesser probability of the consensus being in favor of the innovation, and by the time needed to reach such a consensus. In this respect, one relevant factor in this study is the comparison of probabilities and times needed for consensus under various organizational o market structures, including Hierarchical graphs, lattice graphs, Erdös-Rényi (E.R.) networks [19], Scale-free networks [20], Stars and Complete graphs.

The results of the simulations show that the hierarchy is the formal structure that gives greater probabilities of reaching a consensus in favor of innovation. This is the case for practically the whole range of possible differences in the economic value attributed to innovation with respect to the status quo, and for both large and small-sized social structures. The differences are greater when the relative performance of the innovation on the status quo is not very large. Nevertheless, despite this higher probability of success regarding other topologies, hierarchy is the structure that requires more time to reach a favorable consensus. As is well known, time has a cost (referred to in economy as opportunity cost), established by the profitability of its alternative use. On the other hand, the hierarchical appears as a very stable structure, showing a lower sensitivity to changes in the parameters of the model. ER and BA structures give a very low probability of adoption for small innovation performance with respect to the status quo and are especially sensitive to changes in the rate of learning and social pressure. However, they present an remarkable advantage in the time needed to achieve favorable consensus as compared with a hierarchical graph.

## The model

Let ℐ = {1, 2, …, *N*} be the set of nodes in a social system structure: each node represents an individual or a team of several individuals under a common working method (whether subject to discipline or reached by consensus). For each node *j* ∈ ℐ there is, at least, another node *i* ∈ ℐ,*i* ≠ *j* to which it is connected; the graph of relationships is specified by a (*N* × *N*) symmetric adjacency matrix **A** defined as *A*_*ij*_ = 1 whenever *i* is directly connected to *j* in the social structure, or *A*_*ij*_ = 0 otherwise. We define the influence set of node *j* as the collection of nodes that are directly connected to it: *H*_*j*_ = {*i* ∈ ℐ∣*A*_*ij*_ = 1}. In the same way, the degree *k*_*j*_ of node *j* is the number of nodes directly connected to it: $k_{j}=card(H_{j})=\sum_{i=0}^{N}A_{ij}$.

Each node *i* ∈ ℐ has knowledge of each working method. Let $R_{t}^{i}$ be the performance of node *i* at time *t* provided by the conventional method (status quo) and, in the same way, let $R_{t}^{*i}$ be the node *i*’s performance provided by the new method (innovation). In addition, each node *i* is characterized by a variable *s*_*i*_ (the *strategy*) that takes the value $s_{t}^{i}=1$ if the node *i* supports the innovation at time *t*, or $s_{t}^{i}=0$ otherwise. Let $a_{t}^{i}$ be the number of nodes connected to *i* that support the innovation at time *t*, and let $b_{t}^{i}$ be the number of *i*’s neighbors that support the status quo:

$$\begin{array}{ccc} a_{t}^{i} & = & \sum_{j=1}^{N}s_{t}^{j}A_{ij}.\\ b_{t}^{i} & = & \sum_{j=1}^{N}\left|1-s_{t}^{j}\right|A_{ij}=k_{i}-a_{t}^{i} \end{array}$$ (1)

Given two nodes (*i*, *j*) mutually connected, *i* having adopted the innovation and *j* having not (*i.e.*, $s_{t}^{i}=1,s_{t}^{j}=0$), we establish that they can interact with each other by exchanging knowledge (and exchanging methods) only if the performance difference between the two nodes is contained in an interval *ϵ*:

$$\begin{array}{c} |R_{t}^{*i}-R_{t}^{j}|<\epsilon \;\;, \end{array}$$ (2)

resulting in mutual learning. When condition 2 is satisfied, we define the link (*i*, *j*) as feasible. This threshold *ϵ* introduces a condition for mutual benefit: while high values of *ϵ* allow altruism (*i.e.*, people with high performance help people with very low performance without expecting anything in return), low values of *ϵ* only allow win-win relationships.

### Initial conditions

The system begins with a number *ρ* of randomly distributed nodes (the initial adopters) that adopt the innovation from the first moment *t* = 0, while the other nodes are supporters of the status quo. The performance provided by the status quo is randomly distributed between the nodes according to a gaussian distribution centered in *R* = 1 with variance *σ*. Only the initial adopters obtain more performance from the new method, for these nodes the performance provided by the innovation is distributed according to a gaussian centered in R* with variance *σ*, while for the rest of the nodes (*i.e.*, the supporters of the status quo) the efficiency of both methods is the same. Then, the quotient R*/*R* represents the innovation performance value (*i.e.*, the innovation technical superiority).

Explicitly, at *t* = 0, each node *i* ∈ ℐ, with probability *ρ*/*N*, adopts the innovation ($s_{t=0}^{i}=1$) and its performances are randomly assigned according to the following distributions:

$$\begin{array}{ccc} p(R_{t=0}^{i}=z) & = & \frac{1}{\sigma \sqrt{2\pi }}e^{-\frac{{\left(z-1\right)}^{2}}{2\sigma^{2}}}\;,\\ p(R_{t=0}^{*i}=z) & = & \frac{1}{\sigma \sqrt{2\pi }}e^{-\frac{{\left(z-R^{*}\right)}^{2}}{2\sigma^{2}}}\;. \end{array}$$ (3)

Otherwise, that is, with probability 1−(*ρ*/*N*), node *i* supports the status quo ($s_{t=0}^{i}=0$) and its performances are given by:

$$\begin{array}{c} p\left(R_{t=0}^{i}=z\right)=\frac{1}{\sigma \sqrt{2\pi }}e^{-\frac{{\left(z-1\right)}^{2}}{2\sigma^{2}}}\;,\;\;\;\;\;\;R_{t=0}^{*i}=R_{t=0}^{i}\;. \end{array}$$ (4)

### Dynamics

At each dynamical step, each node chooses (if available) a random feasible neighbor with opposite strategy, such that a given node interacts only once at most per step. Then, a time step consists of at most *N*/2 interactions, and each interaction takes place between two nodes (*i*, *j*) with different strategies (without loss of generality, $s_{t}^{i}=1,s_{t}^{j}=0$). During the process of learning each node shares information on the adopted method, so that a node may improve its performance on the non-adopted method, provided that the opposite node has greater performance on it. The increment is proportional to the difference in performances:

$$\begin{array}{ccc} R_{t}^{*j} & = & R_{t}^{*j}+m\left(R_{t}^{*i}-R_{t}^{*j}\right)\;H\left(R_{t}^{*i}-R_{t}^{*j}\right)\;,\\ R_{t}^{i} & = & R_{t}^{i}+m\left(R_{t}^{j}-R_{t}^{i}\right)\;H\left(R_{t}^{j}-R_{t}^{i}\right)\;, \end{array}$$ (5)

where *m* is a parameter that models the learning ratio by modulating the performance increment resulting from the information exchange: the higher *m*, the greater the increment in performance. *H* is the Heaviside function that takes the value *H*(*a*) = 1 when *a* > 0, and 0 otherwise.

After the learning process, node *j* imitates the strategy of *i* (*i.e.*, *j* adopts the innovation) with probability:

$$\begin{array}{c} P_{t}^{j\leftarrow i}=\frac{\left(1+\delta b_{t}^{j}\right)R_{t}^{*j}}{\left(1+\delta a_{t}^{i}\right)R_{t}^{i}+\left(1+\delta b_{t}^{j}\right)R_{t}^{*j}} \end{array}$$ (6)

where *δ* is a parameter that represents the social pressure: the higher the value of *δ*, the greater the influence exercised by the neighbors. Otherwise, node *i* imitates *j*’s strategy, which implies that the probability for node *i* to adopt the status quo is given by:

$$\begin{array}{c} P_{t}^{i\leftarrow j}=1-P_{t}^{j\leftarrow i}=\frac{\left(1+\delta a_{t}^{i}\right)R_{t}^{i}}{\left(1+\delta b_{t}^{j}\right)R_{t}^{*j}+\left(1+\delta a_{t}^{i}\right)R_{t}^{i}} \end{array}$$ (7)

### Topologies

In order to model different types of organizations and social system’s structures, we consider several network topologies that represent the nodes and connections of the system being simulated. In particular, we have dealt with the following types of graphs:

- **Hierarchical graphs** are networks in which any two nodes are connected by exactly one simple path. In this work, we will use regular hierarchical graphs, in which any intermediate node has a *higher-level* neighbor (its upper-neighbor) and *M* *lower-level* neighbors (its lower-neighbors). Therefore, the degree of an intermediate node *i* is *k*_*i*_ = *M*+1. In addition, leaf nodes do not have lower-neighbors but only upper-neighbor (*k*_*l*_ = 1), and one node (the top-node) does not have upper-neighbor but only lower-neighbors (*k*_*b*_ = *M*). The number of lower-neighbors *M* is called degree of branching.
- In **lattice graphs** the nodes are disposed in the vertex of a tiling and connected to the *k* closest nodes. We will use square lattices with constant degree *k*_*i*_ = 4, ∀ *i* ∈ ℐ. The characteristic distance between two nodes is proportional to the network size.
- **Erdös-Rényi (ER) networks** are random graphs in which the probability *p* for any pair of nodes to be connected is uniform. They are characterized by a binomial degree distribution $P(k_{i}=k)=\left(\begin{array}{c} N-1\\ k \end{array}\right)p^{k}{\left(1-p\right)}^{N-1-k}$, which tends to a Poisson distribution for large networks $P(k_{i}=k)\to \frac{{\left(Np\right)}^{k}{\text{e}}^{-Np}}{k!}$. [19]
- **Scale-free networks** are graphs whose degree distribution follows a power law *P*(*k*_*i*_ = *k*) = *ck* ^−*γ*^, resulting in a small number of highly connected (*i.e.*, very influential) nodes: the hubs. In particular, we will use the Barabási-Albert (hereafter, BA) algorithm [20] to generate the networks.
- **Stars** are particular cases of hierarchical graphs without intermediate nodes. They consist of a central node (the hub, *k*_*hub*_ = *N*−1) connected to all other nodes, which have only that connection (*k*_*i*_ = 1).
- **Complete graphs** are networks in which every pair of nodes are directly connected by a link. The degree of any node is *k*_*i*_ = *N*−1

## Results

According to section *Initial conditions*, *ρ* initial adopters were randomly distributed among a population of *N*−*ρ* supporters of the status quo. After that, the dynamics of the adoption process was run. The simulations were stopped when the number of active nodes (*i.e.*, agents with non-zero probability of changing opinion) vanished. The results shown below were obtained by averaging over a large number (typically 10^4^) of different initial conditions and network realizations.

**Fig 1:**
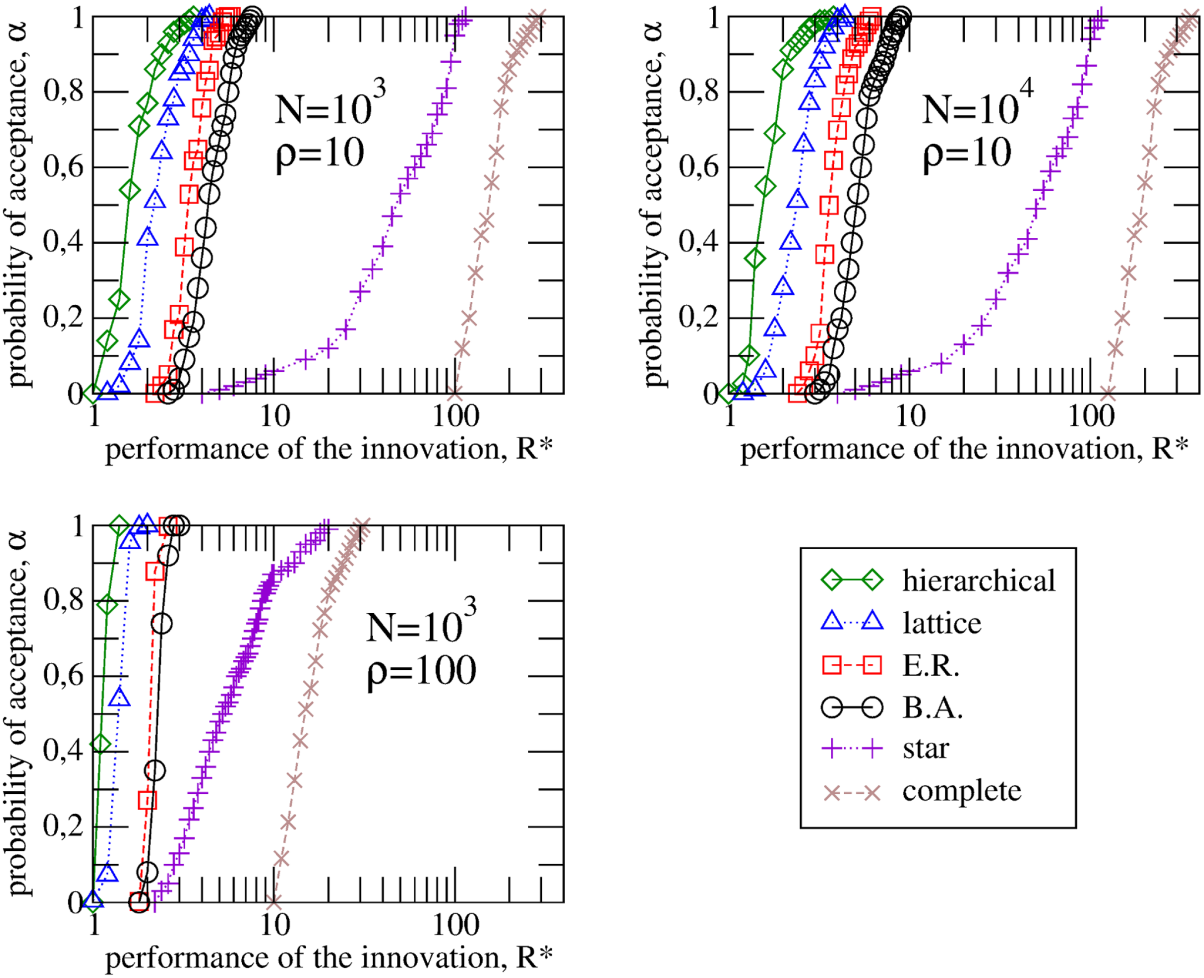
Acceptance probability versus the performance of the innovation. Fraction of realizations in which the innovative method has been finally adopted as a function of the initial performance of the new method R* for six different types of networks: hierarchical, lattice, Erdös-Rényi, Barabási-Albert, star and complete. Left panels correspond to systems of *N* = 10^3^ agents while the right panel to *N* = 10^4^. Upper panels correspond to the case of a number of initial adopters *ρ* = 10, while the bottom panel to *ρ* = 100. Other values are *R* = 1, *δ* = 0.5, *m* = 0.5, *ϵ* ≫ R*, *σ* = *N* ^−1^. Each point is averaged over 10^4^ network realizations.

Global consensus is finally reached for all the topologies studied, that is, for each simulation, the innovation is either accepted or rejected by all the agents bringing the system to a frozen state. Consequently, for a given set of parameter values, the fraction of realizations *α* in which the innovative method has been finally adopted represents the probability of adoption for the innovative method or technology. In Fig 1, we plot this fraction *α* versus the average initial performance of the new method R*, where each point represents the numerical result averaged over 10^4^ different network realizations. Without loss of generality, the average initial performance of the status quo was fixed to *R* = 1, therefore, R*/*R* = R*, which means that R* represents the improvement in performance of the new method. Different symbols and curves refer to the different structures studied: hierarchical, lattice, ER, BA, star and complete. Other parameters are indicates in the figure.

Firstly, to study the influence of the degree distribution on the dynamics, let us focus our attention in the first four topologies: hierarchical, lattice, ER and BA, whose networks realizations were constructed with the same mean connectivity ⟨*k*⟩ ∼ 4. As shown, the regular graphs (hierarchical and lattice) show higher acceptance probabilities than the complex networks (ER and BA) and, in turn, ER networks show higher success probabilities than BA graphs. In conclusion, for a given mean connectivity, increasing degree heterogeneity decreases the likelihood that the proposal will be accepted. Furthermore, comparing the two top panels, it can be observed that the size of the system does not have a significant influence on the acceptance probability. Nevertheless, in heterogeneous and star graphs, large system sizes show lower ratios of acceptance. Regarding the effect of the seed size, as can be seen by comparing the curves in the bottom panel which those shown in the up-left panel, the increase of the number of initial adopters has a positive influence on the success probability. Comparing the three panels, we see that it is the amount of initials adopters *ρ*, not its fraction, that determines the success probability. This is due to the fact that achieving a critical mass of adopters in early stages is key to the success of the innovation. This result has important implications because it indicates that the efforts required to spread an innovation are independent of the size of the system.

**Fig 2:**
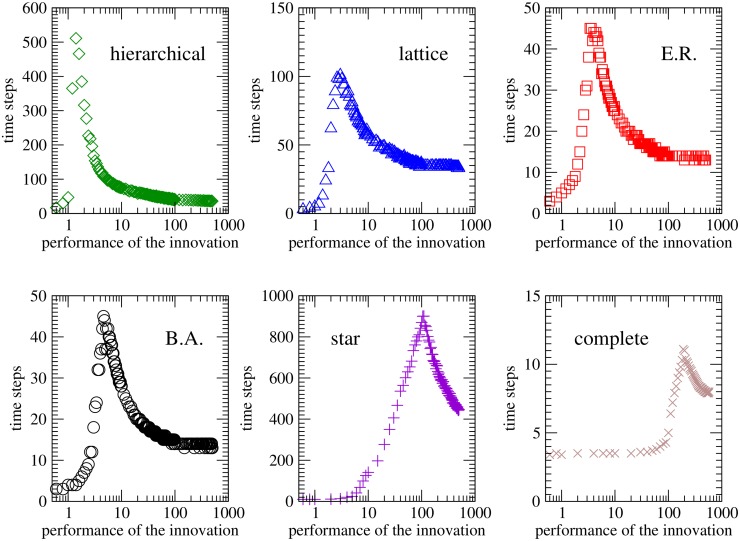
Consensus time versus versus the performance of the innovation. Number of steps needed to reach a consensus (either to accept or reject the innovation) as a function of the the initial new method’s performance for the six different topologies considered. By comparing this panels from the curves shown in Fig 1, it can be observed that the values of R* corresponding to the maximum consensus times match the values of R* for which *P*(*acceptance*) ∼ 0.5. Other values are *N* = 1000, *R* = 1, *ρ* = 10, *δ* = 0.5, *m* = 0.5, *ϵ* ≫ R*, *σ* = *N* ^−1^. Each point is averaged over 10^4^ network realizations.

Each panel of Fig 2 represents, for each of the different topologies considered, the time needed to reach global consensus, that is, the number of dynamical steps until all the agents of the system shared the same opinion about the innovation. For each structure of the networks of contacts we see how a peak in the transition time is revealed, signaling the existence of a phase transition between the two different final states. In fact, by comparing these results with those shown in the upper-left panel of Fig 1, it can be observed that the values of the innovation performance R* corresponding to the maximum consensus time (the peaks of Fig 2) match the values of R* for which the acceptance probability has intermediate values (transitions in Fig 1). This is because, while for low values of R* the proposal is rejected in the initial phases and for high values of R* it is accepted in most interactions, for values of R* close to the transition the probability for individuals to accept the proposal takes intermediate values, causing fluctuations which in turn slow down the convergence to the final state.

**Fig 3:**
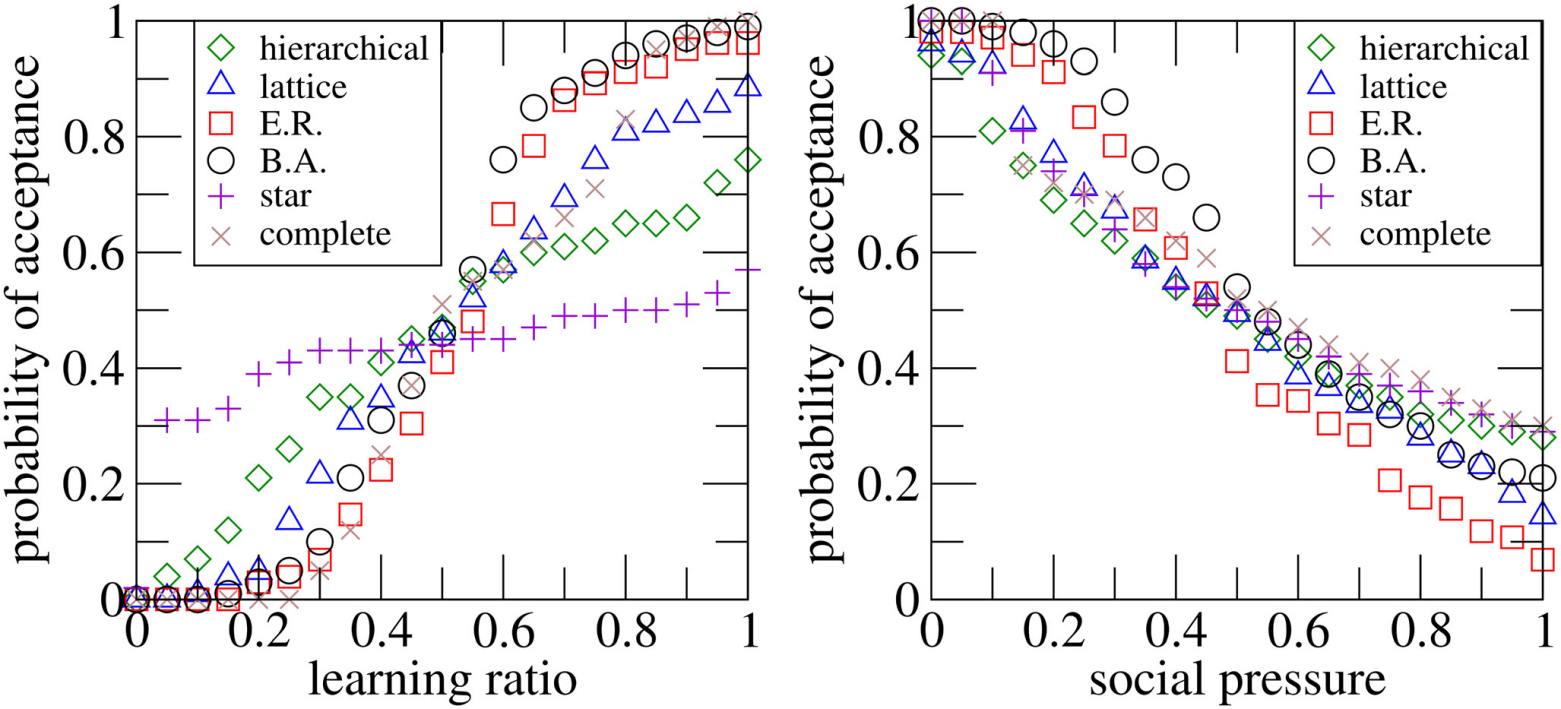
Acceptance probability versus learning ratio and social pressure. Fraction of realizations in which the innovation has been adopted *P*(*acceptance*) versus the learning ratio *m* (left panel) and versus the social pressure parameter *δ* (right panel) for the six different types of networks studied. The value of R* is chosen so that *P*(*acceptance*) ∼ 0.5 for *m* = 0.5, being R* = 1.55, 2.2, 3.3, 4.5, 35, 155 for the hierarchical, lattice, Erdös-Rényi, Barabási-Albert, star and complete graphs respectively. Other values are *N* = 1000, *R* = 1, *ρ* = 10, *δ* = 0.5, *ϵ* ≫ R*, *σ* = *N* ^−1^. Each point is averaged over 10^4^ different realizations. See the main text for further details.

Regarding the effect of the learning process on the opinion dynamics, the left panel of Fig 3 represents the acceptance probability *P*(*acceptance*) versus the learning ratio parameter *m* for the six different topologies considered. According to the results showed in Fig 1, the initial performance of the innovation R* for each topology is chosen so that *P*(*acceptance*) ∼ 0.5 for *m* = 0.5, being R* = 1.55, 2.2, 3.3, 4.5, 35, 155 for the hierarchical, lattice, Erdös-Rényi, Barabási-Albert, star and complete graphs respectively. As shown, the more information exchange, the greater the likelihood of acceptance of the innovation. The first four kinds of networks (hierarchical, lattice, ER and BA) were made up with the same mean connectivity ⟨*k*⟩ ∼ 4. Among these topologies, regular networks (hierarchical and lattice) show smoother transitions than complex graphs (ER and BA), which means that degree heterogeneity increases the sensitivity to the learning process, while regular structures are more robust. Furthermore, star structures are less sensitive to the learning ratio. On the other hand, with respect to the influence of social pressure on the dynamics, the right panel of Fig 3 represents, for the same initial performance values R*, the fraction of realizations in which the innovation has been adopted as a function of the social pressure parameter *δ* for the different social structures considered. As can be observed, social pressure makes it harder for the innovation to spread regardless of the structure of the network, which is a consequence of the fact that, according to Eqs 6–7, the imitation probability decreases with increasing social pressure.

**Fig 4:**
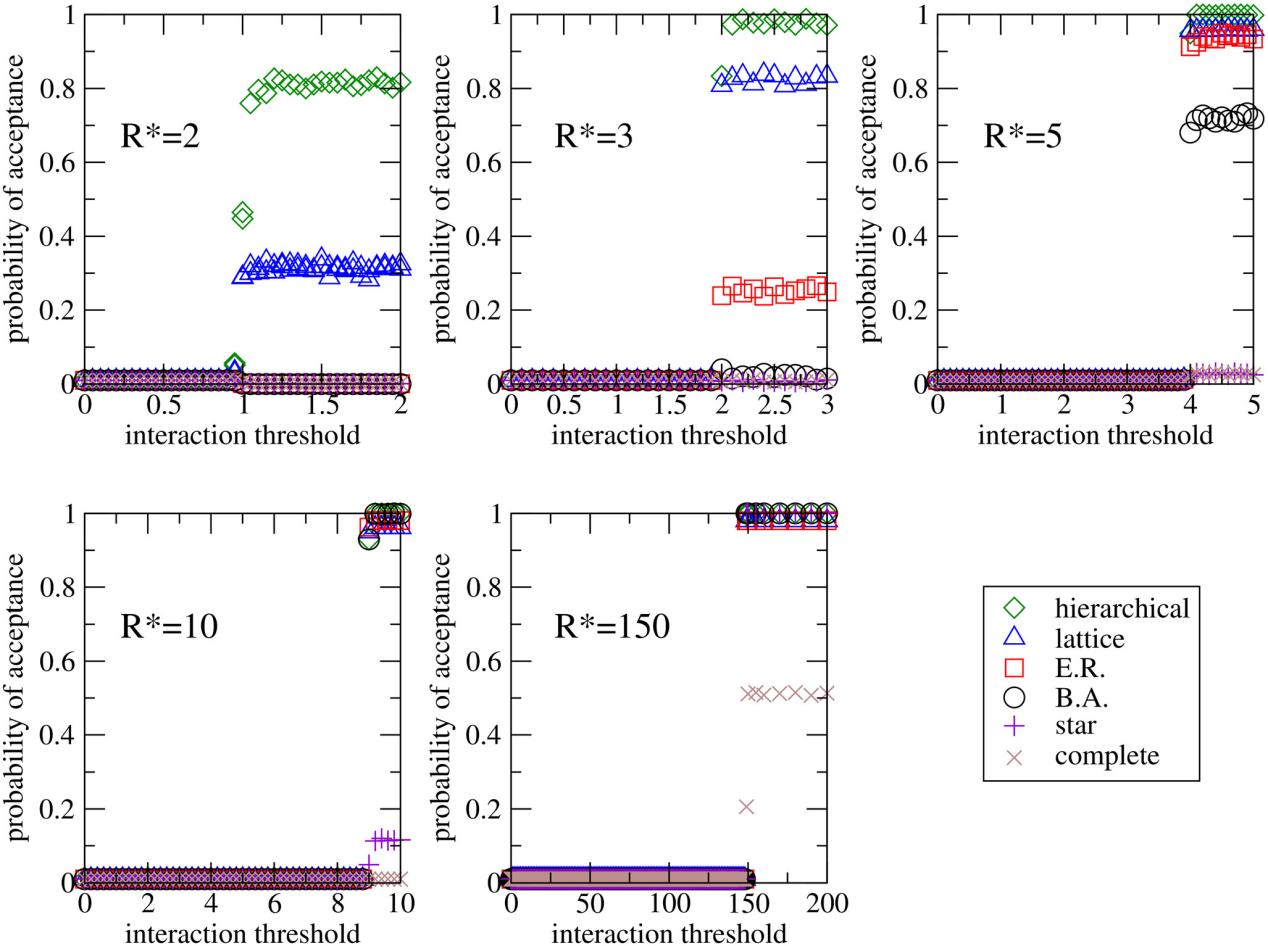
Acceptance probability versus the interaction threshold. Fraction of realizations in which the innovation has been adopted as a function of the performance difference threshold *ϵ* beyond which two nodes do not interact with each other by exchanging knowledge and exchanging methods, for the six different topologies studied and different values of the initial performance of the innovation R* = 2, 3, 5, 10, 150. Other values are *N* = 1000, *R* = 1, *ρ* = 10, *δ* = 0.5, *m* = 0.5, *σ* = *N* ^−1^. Each point is averaged over 10^4^ network realizations.

Let us now focus on the impact of restrictions on interactions between agents. According to formula Eq (2), two agents can interact with each other by exchanging knowledge and methods only if a principle of minimal trust is satisfied, namely, if the absolute value of the performance difference between both agents is lower than a threshold *ϵ*. Fig 4 represents the probability of acceptance of the innovation as a function of the parameter *ϵ*; each panel plots the results for a given value of the innovation performance value R*, while each symbol represents each of the different topologies considered. As can be observed, all the curves are consistent with a step function centered at R*−*R* = R*−1, which means that, although individual performances ($R_{i},R_{i}^{*}$) may vary due to the learning process, the threshold *ϵ* has no influence on the final state provided that the minimum trust principle is satisfied at the initial state.

**Fig 5:**
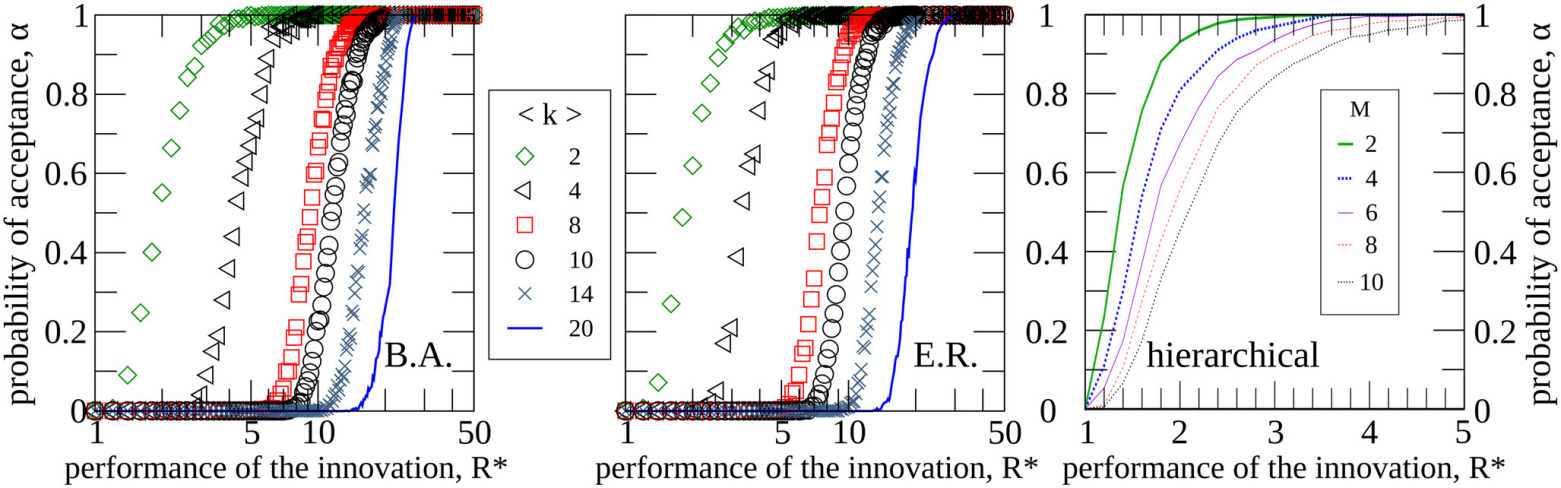
Influence of the connectivity on the acceptance probability. Fraction of realizations in which the innovative method has been adopted versus the initial performance of the innovation R* for different values of the mean connectivity ⟨*k*⟩ (left and center panel) and for different values of the degree of branching *M* (right panel). Left panel corresponds to Barabási-Albert networks, center panel to Erdös-Rényi graphs and right panel to hierarchical structure. Other values are *N* = 1000, *R* = 1, *ρ* = 10, *δ* = 0.5, *m* = 0.5, *ϵ* ≫ R*, *σ* = *N* ^−1^. Each point is averaged over 10^4^ network realizations.

Regarding the effect of connectivity on the opinion dynamics, the left and center panels of Fig 5 show the acceptance probability as a function of the initial performance of the innovative method for different values of the mean connectivity ⟨*k*⟩. The left panel corresponds to Barabási-Albert graphs and the center panel to Erdös-Rényi networks. As shown, increased connectivity hinders the diffusion of the innovation, which is a consequence of the fact that social pressure increases with increasing the number of contacts and therefore, in the first states, the probability for an agent to accept the innovation. In the same way, the right panel of Fig 5 studies the influence of the degree of branching *M* (*i.e.*, the number of lower-neighbors of an intermediate node) on the acceptance probability in the hierarchical structures. The curves show the fraction of realizations in which the innovative method has been adopted as a function of the initial new method’s performance R* for different values of *M*. As illustrated in the figure, increasing the degree of branching implies a decrease in the probability of the new method being adopted, as a consequence of the increase in social pressure caused by the increase of contacts.

## Discussion

Although the main aim of this work is to study the dynamics of the diffusion of innovations, this paper can be useful for understanding the adoption as a problem of opinion formation in human groups. The diffusion of innovations in markets takes time because not all individuals adopt at the same time, where adoption means that individuals purchase or use the innovation. Within the organization, when the adoption of an innovation involves the generalized use of it among all members the diffusion process will be affected by how the collective decision process is structured and managed. The literature on public opinion [21–23] describe this *forming* as the result of a process of influences of some people over others, using unidirectional means of influence (for example, mass media) or multiple directional ones (for example, social networks). In some scenarios all individuals have the same capacity to exert influence while in others there are opinion leaders with a greater level of influence than anyone else [24].

According to this approach, this paper belongs to the studies that analyze the dissemination process of an opinion, using computer simulation of mathematical models of interpersonal influences in networks with nodes and lines of communication linking these nodes. In the context of our work the opinion-formation ends up building a consensus, whether favorable or not, around an innovation that arises at some particular points in the organization. Each person in the organization has attached a likelihood of accepting the innovation which increases with the *positive externalities* (which are attributed to network effects [25], coordination games [26], learning from others [27], social pressure [28] and trust [29]) resulting from the pressure to adopt a favorable opinion exerted by the members that had opted for that favorable position previously, capability to learn about de alternatives, and with economic value of the innovation. Our paper uses the same methodology of simulating mathematical models of interpersonal influences as [30] on public opinion formation. The authors assume that some individuals have different influence than the others (opinion leaders and followers); the probability of staying to one opinion is either zero or one; no change of opinion is contemplated; and the networks that determine the mutual influences are formed at random. In our study, all individuals are equal (although the model can incorporate influential asymmetries); the probabilities of supporting one opinion or another are between zero and one; people can change their status, either for or against, between one iteration and the next; the relative value of the innovation is included as a determining factor for the likelihood of support; people learn from others about economic value of alternatives giving heterogeneity; and the networks in which diffusion occurs respond to different structures commonly found in the market o real organizations as business firms. So far organizations and organization structures are viewed as institutions for solving coordination and motivation problems [31], and as tools for creating, transferring and using knowledge [32]. Our paper also demonstrates the relevance of the formal structure of a social system in enabling the assimilation of proposals for change and innovative initiatives, *i.e.* as determinant of social systems’ innovation capacity.

## Summary and concluding remarks

The diffusion of innovation, that is, the study of patterns of how new ideas or technologies spread throughout a community is a topic of interest in many fields, including economics, sociology, market research and politics. In this paper we have studied the probability for a proposal to be accepted by different collectives. Different communities are modeled through different topologies of the contact network, and the process is studied through an agent based model whose inter individual interactions mimic both the learning process and the acceptance or rejection of the proposal.

Our results show that the structure of the network of contacts has a strong influence on the innovation diffusion, being more difficult for a proposal to be accepted when the connectivity of agents is heterogeneously distributed. We have shown that the learning process plays a positive role in the diffusion, being heterogeneous structures more sensitive to the lack of information exchange. We have also studied the effect of social pressure on the acceptance dynamics, showing that social pressure hinders innovation spreading irrespective of the collective structure. Finally, we have shown that networks with high average connectivity obstruct the diffusion of innovation.

These results are of interest for understanding how different factors influence the diffusion and acceptance of a technological, technical or legislative proposal in different communities.
